# Comparison of shear bond strength of orthodontic brackets bonded with a universal adhesive using different etching methods

**DOI:** 10.1590/2177-6709.24.4.33.e1-8.onl

**Published:** 2019

**Authors:** Fereshteh Shafiei, Ahmadreza Sardarian, Reza Fekrazad, Amin Farjood

**Affiliations:** 1 Shiraz University of Medical Sciences, School of Dentistry, Oral and Dental Disease Research Center (Shiraz, Iran).; 2 Shiraz University of Medical Sciences, School of Dentistry, Orthodontics Research Center (Shiraz, Iran).; 3 AJA University of Medical Sciences, School of Dentistry, Laser research center in medical Sciences (Tehran, Iran).; 4 Universal Scientific Education and Research Network, International Network for Photo Medicine and Photo Dynamic Therapy (Tehran, Iran).; 5 Shiraz University of Medical Sciences, School of Dentistry, Department of Orthodontics (Shiraz, Iran).

**Keywords:** Orthodontic adhesive, Orthodontic brackets, Laser

## Abstract

**Objective:**

The aim of this study was to compare the effects of three enamel etching modes - laser-etch, self-etch and acid-etch (5, 10 and 15 s) - on bracket bonding, using a universal adhesive.

**Methods:**

Eighty-four maxillary premolars were randomly divided into seven groups (n=12) based on the etching method and the adhesive used for bracket bonding. After water storage and thermocycling, shear bond strength was measured, and adhesive remnant index scores on debonded enamel were determined.

**Results:**

There were significant differences between the seven groups regarding bond strength values (*p*< 0.001). The highest values were observed in universal adhesive with laser etching group, while Transbond XT with acid or laser etching, and universal adhesive used in self-etch mode demonstrated the lowest bond strength. The universal adhesive with the three different etching times presented with statistically similar results, all showing an improvement in bond strength, compared with Scotchbond universal (SBU)/SE.

**Conclusions:**

The universal adhesive evaluated in the present study demonstrated statistically similar bond strengths to conventional orthodontic adhesive in self-etch mode. The bond strength can be improved by adding an initial acid etching or laser conditioning step, although enamel damage was observed in some cases.

## RESUMO

**Objetivo:**

o objetivo desse estudo foi comparar os efeitos de três métodos de condicionamento do esmalte - condicionamento a *laser*, autocondicionamento e condicionamento ácido (5, 10 e 15 segundos) - sobre a colagem de braquetes com um adesivo universal.

**Métodos:**

oitenta e quatro pré-molares superiores foram divididos aleatoriamente em sete grupos (n=12) de acordo com o método de condicionamento e o adesivo utilizado para a colagem dos braquetes. Após armazenamento em água e termociclagem, a força de colagem foi medida e o índice de adesivo remanescente no esmalte após a descolagem foi determinado.

**Resultados:**

houve diferenças significativas entre os sete grupos com relação aos valores de força de colagem (*p*< 0,001). Os valores mais altos foram observados no grupo com adesivo universal e condicionamento a *laser*, enquanto o Transbond XP condicionado com ácido ou *laser,* e o adesivo universal autocondicionante demonstraram as menores forças de colagem. O adesivo universal apresentou resultados estatisticamente semelhantes para os três diferentes tempos de condicionamento ácido, todos mostrando um aumento na força de colagem, em comparação ao Scotchbond Universal.

**Conclusões:**

o adesivo universal avaliado no presente estudo demonstrou força de colagem estatisticamente semelhante aos adesivos ortodônticos convencionais do tipo autocondicionante. A força de colagem pode ser aumentada acrescentando-se uma etapa inicial de condicionamento ácido ou com *laser*, embora danos ao esmalte tenham sido observados em alguns casos.

## INTRODUCTION

For many years phosphoric acid etching has been widely used as the main step of bonding orthodontic brackets. The differential dissolution of enamel crystals and the resultant roughened surface is known to be responsible for successful micromechanical bonding to enamel.^1^ However, this approach takes a long time, especially in the initial set up session, which is associated with patient discomfort. Loss of surface enamel following acid etching has been reported with values ranging from 0.2 to 25 µm, depending on etching duration, acid concentration and structure/composition of enamel. Furthermore, acid etching creates a morphologically porous layer 5-50 µm deep, which could render the teeth susceptible to staining.^2^ ^,^ ^3^ Moreover, increased decalcification and white spot formation around bonded brackets during orthodontic treatment have also been reported as possible disadvantages of the conventional bonding technique.^2^ ^,^ ^4^ ^,^ ^5^ Enamel damage might occur during bracket removal and elimination of the high bulk of residual adhesive resin.^6^ These shortcomings interfere with the primary goal of the treatment in improving esthetics and appearance of the teeth, leading to research efforts in order to find suitable alternatives. 

Laser etching has been used in numerous studies and has been shown to render comparable,^7^ ^,^ ^8^ higher^9^ and lower^4^ ^,^ ^10^ bond strength values, compared with that of acid etching, depending on the laser type, irradiation parameters and experimental designs. An increase in calcium-to-phosphorous ratio^10^ and the resultant acid/caries resistance of laser-irradiated enamel surface could be attractive advantages^4^ ^,^ ^11^ of the laser etching method. Er,Cr:YSGG is a relatively new laser and has been demonstrated to be the safest and most effective hard tissue laser.^8^ A study in the field of restorative dentistry has suggested that composition of the adhesive might affect bond strength to laser-irradiated enamel in composite resin restorations.^12^

In addition to enamel preparation, adhesive/bonding resins affect the bracket bonding to enamel.^1^ Self-etch (SE) or etch-and-dry adhesives are less aggressive on enamel, rendering advantages such as easy application, saving time and with lower risk of contamination by saliva during bracket bonding.^13^ ^,^ ^14^ Despite restricted penetration of this adhesive into superficial enamel with shorter resin tags,^13^ some studies demonstrated promising results for simplifying bracket bonding.^1^ ^,^ ^13^ ^,^ ^14^ Furthermore, it has been reported that in bracket debonding, the chance of enamel damage is decreased, with fast and easy removal of residual resin.^6^

Recently, a new type of single-step one-bottle universal adhesive (UA) has been introduced into restorative dentistry, which has been claimed to present the ability to bond with numerous surfaces (enamel, dentin, amalgam and porcelain). According to the manufacturers, these adhesives have a unique ability to be used in both etch-and-rinse and self-etch modes.^15^ However, inadequacy of self-etching approach on enamel surface has been indicated by some authors, who have recommended phosphoric acid etching for 15 or 30 s to obtain high enamel bond strength.^16^ ^,^ ^17^ Nevertheless, shortened etching times, as low as 3 s, have been reported as sufficient by a recent study.^18^

To date, only one study in the field of restorative dentistry has evaluated the bond strength of UAs to enamel in the three modes (acid etch, self-etch and laser etch), reporting comparable strength for self-etching and Er,Cr:YSGG laser etching, and higher strength for pre-acid etching.^19^

No study has been published on bracket bonding using UAs with different surface preparations along with shortened acid etching time. Therefore, this study was designed to examine the null hypothesis that different etching modes and different acid etching times (less than 15 s) would not affect bracket bonding using a universal adhesive.

## MATERIALS AND METHODS 

Eighty-four maxillary premolars with intact buccal surfaces, which were extracted for orthodontic purposes, were selected for this study and stored in 0.5% chloramine-T solution for two weeks, to ensure disinfection. Absence of cracks and defects in the teeth was verified under a stereomicroscope (Carl Zeiss, Oberkochen, Germany). The buccal surfaces of the teeth were cleaned using a rubber cap and slurry of non-fluoridated pumice.

The teeth were vertically mounted in self-cured acrylic resin cylinders and were randomly divided into seven groups (n=12), according to surface treatment procedures.

» Group 1 (etch/TXT): As control, phosphoric acid etching for 30 s, water rinsing for 15 s, air-drying for 10 s and application of Transbond XT primer ( Lot #N704516,3M, Unitek, Monrovia, CA).
» Group 2 (laser/TXT): Er,Cr:YSGG laser etching using WaterLase Plus/Gold handpiece (Biolase technology Inc, Cromwell Irivine, CA, USA) with a wavelength of 2780 nm; tip type MZ8; pulse duration 60 µs; repetition rate 50 HZ; and power 2 W, for 10 s at a distance of 1 mm and perpendicular to the enamel surface with water and air spray. Transbond XT primer was applied.
» Group 3 (etch-15/SBU): acid etching, water rinsing for 15 s and air drying for 10 s and application of Scotchbond Universal (SBU) adhesive (3M ESPE, St Paul, MN, USA).
» Group 4 (etch-10/SBU): acid-etching for 10 s, rinsing for 15 s, drying for 10 s and application of SBU adhesive.
» Group 5 (etch-5/SBU): acid etching for 5 s, rinsing for 15 s, drying for 10 s and application of SBU.
» Group 6 (SE/SBU): air drying and application of SBU (SE mode) which was gently rubbed for 20 s on the tooth surface.
» Group 7 (laser/SBU): laser etching similar to Group 2 and application of SBU.

To standardize distance and area of laser irradiation in Groups 2 and 6, acrylic discs (1 mm in thickness with a 3×4 mm hole in the center) were used.

After performing the corresponding surface treatments, stainless steel maxillary premolar brackets (American Orthodontics, Sheboygan, WI) with a bracket base area of 8.82 mm^2^ were bonded with light-cured orthodontic adhesive composite resin (Transbond XT) at the center of the clinical crown. The adhesive resin was applied to the base of the bracket, and then the bracket was pressed firmly onto the prepared enamel surface. Excess adhesive was removed from bracket margin using a scaler. In the SBU groups, before application of the adhesive, a thin layer of SBU was applied to the bracket base, air dried and light-cured for 10 s. This step was added to take advantage of the enhanced bond of SBU with metallic surfaces. This step and light-curing were performed in the same manner by one experienced specialist. Light was applied for 40 s (10 s from each side) at distance of 1-2 mm of light tip from bracket margins using a portable light curing device (LITEX 696 Cordless LED Curing Light, Dentamerica, San Jose, CA). The samples were stored in 37°C distilled water and then thermocycled for 3000 cycles between 5°C and 55°C with a dwell time of 30 s.

Bracket bond strength was tested using a chisel edge blade at the bracket/enamel interface, in a universal testing machine (Zwick/Roell, Z020, Ulm, Germany), in such a way that the bracket base was parallel to the direction of shear loading, creating shear force at the bracket-enamel interface at a crosshead speed of 0.5 mm/min, with a load cell of 2 kilonewton. The debonding force was recorded in Newton and converted into MPa.

After debonding, the enamel surfaces were subjected to stereomicroscope evaluation (×20 magnification) to determine the amount of remaining adhesive on the enamel, by a blinded operator according to the adhesive remnant index (ARI) categories, as follows:

0 = no adhesive remaining;
1 = less than 50% of the adhesive remaining;
2 = more than 50% of the adhesive remaining;
3 = the whole adhesive remaining, showing bracket base impression^14^.

To calculate any possible error involved in the ARI scoring, two groups of the samples (Groups 2 and 4) were selected randomly to be scored again by the same observer, six months after the initial scoring. Exactly the same scores were achieved in the second scoring session. Subsequently, the exposed enamel surface was examined for any enamel cracks under a stereomicroscope. As the data for bond stress demonstrated a normal distribution according to the Kolmogorov-Smirnov test, they were analyzed by one-way ANOVA and *post-hoc* Tukey tests; ARI scores were analyzed with Kruskal-Wallis test, as the data was not continuous and did not have normal distribution.

One debonded tooth from each group was randomly selected for SEM evaluation. The separated coronal parts of the specimens were dehydrated using a desiccator for 24 h and mounted on aluminum stubs using a double-faced carbon tape. Then, the specimens were sputter-coated with gold and observed under the microscope (Tescan, Vega III, England), with an accelerating voltage of 15 Kv.

## RESULTS

The mean bracket bond strengths and standard deviations of the seven groups are presented in Table 1. Kolmogorov-Smirnov test revealed a normal distribution of data for all the groups. According to one-way ANOVA, there were significant differences among the groups (*p*< 0.001). The highest bond strength was achieved in the laser/SBU group (15.4 ± 3.8), with significant differences from all the other groups (*p*< 0.006). The lowest strength was obtained in conventional etch/TXT, laser/TXT and SE/SBU groups (approximately 7.1-7.5 MPa), with no significant differences between them.

**Table 1:** Shear bond strength between bracket base and enamel, in groups with different surface treatment.

Group*	Shear bond strength (MPa)**	
	Mean	Standard deviation
Etch/TXT	7.60^A^	1.56
Laser/TXT	7.14^A^	3.07
Etch- 15/SBU	11.50^B^	3.75
Etch-10/SBU	11.35^B^	3.18
Etch-5/SBU	11.15^B^	2.15
SE/SBU	7.53^A^	3.40
Laser/SBU	15.38^C^	3.78

*Etch/TXT: phosphoric acid-etching for 30 s + Transbond XT primer; Laser/TXT: Er,Cr:YSGG laser etching (2 W for 10 s) + Transbond XT primer; Etch-15/SBU: acid-etching for 15 s + SBU; Etch-10/SBU: acid etching for 10 s + SBU; Etch-5/SBU: acid-etching for 5 s + SBU; SE/SBU: SBU in self-etching mode; Laser/SBU: Er,Cr:YSGG laser etching (2 W for 10 s) + SBU.

**Groups annotated with different superscript letters demonstrate significantly different results (ANOVA, Tukey *post-hoc* test, *p* < 0.05).

The three etch/SBU groups (5, 10 and 15 s) yielded a significantly higher strength, compared to the SBU/SE group (*p*< 0.03). These etch/SBU groups exhibited comparable bond strength (11.1-11.5 MPa) (*p*> 0.05). The results of multiple comparisons by *post-hoc* Tukey tests are shown in Table 1.

The distribution of ARI scores of the seven groups is shown in Table 2. Statistical analysis of ARI scores with Kruskal-Wallis test revealed no significant differences between the groups (*p*= 0.928). Enamel crack was observed in etch/TXT (n=2), etch-15/SBU (n=2) and laser/SBU (n=3) groups.

**Table 2:** Adhesive remnant index (ARI) scores in groups with different surface treatment.

Group*	ARI scores				P-value**
	0	1	2	3	
Etch/TXT	4	2	1	6	0.928
Laser/TXT	3	2	2	5	
Etch- 15/SBU	4	2	3	3	
Etch-10/SBU	3	4	3	2	
Etch-5/SBU	3	3	4	2	
SE/SBU	4	3	3	2	
Laser/SBU	3	3	1	5	

*Etch/TXT: phosphoric acid-etching for 30 s + Transbond XT primer; Laser/TXT: Er,Cr:YSGG laser etching (2 W for 10 s) + Transbond XT primer; Etch-15/SBU: acid-etching for 15 s + SBU; Etch-10/SBU: acid etching for 10 s + SBU; Etch-5/SBU: acid-etching for 5 s + SBU; SE/SBU: SBU in self-etching mode; Laser/SBU: Er,Cr:YSGG laser etching (2 W for 10 s) + SBU.

**The data were analyzed using the Kruskal-Wallis test.

**Figure 1:**
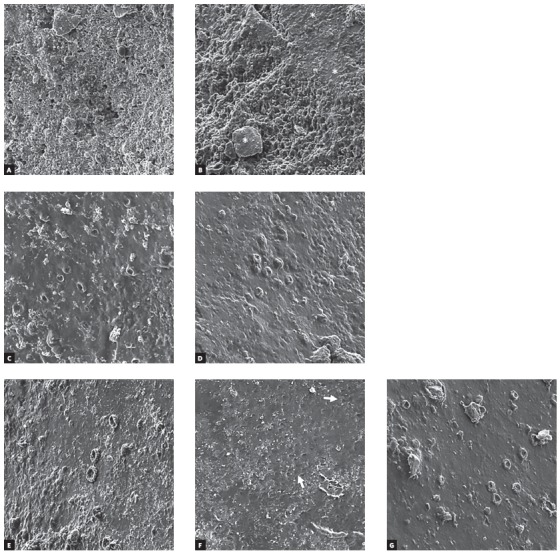
Photomicrograph of the debonded surface in: A) Etch/TXT group, showing microretentive pattern of the enamel surface; B) Laser/TXT group, showing unique rough enamel surface with slight remnant resin (asterisks); C) Etch-15/SBU group, showing the enamel surface covered with resin; D) Etch-10/SBU group, showing the enamel surface covered with resin; E) Etch-5/SBU group, showing the enamel surface covered with resin; F) SE/SBU group, showing less retentive pattern of enamel surface, with some exposed enamel rods (arrows); G) Laser/SBU group, showing the enamel surface homogenously covered with resin.

SEM images obtained after debonding are shown at ×1000 magnification in Figure. As it is evident from the photomicrographs, in TXT/acid-etching or laser etching, the surface with microretentive pattern was observed, while less retentive pattern was seen for SBU in SE mode group. In the SBU/acid-etching groups (Figs 1C, D and E) and the SBU/laser-etching group (Fig 1G), the surface was mainly covered with resin and enamel rods were not visible.

## DISCUSSION

The optimal shear bond strength (SBS) of the bracket to enamel is expected to prevent bracket debonding during treatment, while not causing enamel damage during debonding and keeping the enamel intact after treatment.^20^ It is desirable for clinicians to achieve this bond with an easy and fast approach along with maximum patient comfort. The results of the present study rejected the null hypothesis. Scotchbond Universal (SBU) demonstrated equivalent bond strength to conventional orthodontic adhesives in self-etching mode, and provided superior values when applied after acid and laser etching.

The UA used in this study, SBU with mild acidity (pH=2.7), contains the acidic functional monomer 10-MDP along with vitrebond co-polymer; both are able to interact with hydroxyapatite. The presence of the latter results in less technique sensitivity, regarding moisture contamination.^16^ This property is beneficial in clinical practice for attaching brackets to multiple teeth or in cases where impacted or semi-erupted teeth need to be bonded. SBU in SE mode resulted in comparable SBS to conventional acid etching group. This result was supported by a recent study by Hellak et al.^21^ - however, they measured initial SBS without thermocycling. 10-MDP has been documented to be capable of bonding to enamel and dentin effectively, forming a nanolayer at the adhesive interface. It is composed of calcium salt of MDP with low solubility. In addition, MDP is a relatively hydrophobic monomer.^15^ ^,^ ^22^ These properties might account for reliable bond strength of SBU in SE mode after thermocycling. The bracket-enamel bond was affected by thermocycling due to water sorption and induced thermal stress.^7^

The nanofiller in SBU and the formed thick adhesive layer might induce a beneficial effect in terms of bond strength, via stress relief and cessation of crack propagation, respectively^16^. This positive effect has been demonstrated for filled adhesives in bracket bonding.^23^ Less but adequate SBS of self-etch adhesives compared with conventional etching has previously been reported.^1^ ^,^ ^13^ ^,^ ^14^ ^,^ ^20^ A significantly lower SBS of self-etching primer compared to conventional etching can be attributed to short application time (3 s) of self-etching primer, as shown by Chu et al.^24^ The positive effect of increased application time (15 s) of self-etching primer on SBS of brackets has been documented.^13^ In the current study, SBU was applied and gently rubbed on the surface for 20 s, providing bond strength similar to the conventional orthodontic bonding.

Furthermore, acid etching for 15 s prior to SBU used in this study significantly increased bracket shear bond strength. This difference between SE mode and acid etch mode led to reject the null hypothesis. This confirmed the role of acid etching in creating a porous and retentive enamel surface, in particular on unground enamel involved in bracket bonding. Phosphoric acid is able to remove the less reactive high-mineralized superficial enamel layer.^16^ ^,^ ^25^ Interestingly, reducing acid etching time to 10 s and even 5 s was sufficient to obtain this positive effect of acid-etching, in the present study. Therefore, part of the null hypothesis stating no difference between acid etching times was supported. In self-etch approach, the shallower etching depth and less demineralization of enamel due to lower acidity, and simultaneous etching and adhesive infiltration has been reported. When comparing to acid-etching, SE adhesives create shallower and fewer resin tags.^26^ ^,^ ^27^ The less retentive pattern in the debonded surface of SBU in SE mode group was observed under SEM.

It has been reported that phosphoric acid etching provides a hydrophilic enamel surface by exposing hydroxyl groups of the enamel,^28^ compatible with UAs containing water and hydrophilic monomers.^18^ Moreover, this etching polarizes the enamel surface, improving chemical interaction of acidic functional monomers with hydroxyapatite.^28^ These contributed to increased shear bond performance of SE adhesives and UAs to enamel.^18^ ^,^ ^29^ The modified surface was achieved in shortened etching time (even 3 s) on ground (smear layer-covered) enamel.^18^ ^,^ ^29^

It seems that reducing etching time to 10 and 5 s on intact enamel, as it was the case in our study, was able to provide the above-mentioned beneficial effects to some extent. Previously, lack of a determinant factor for enamel condition (ground and unground) for bonding ability of SE and total-etch adhesives has been reported.^25^ ^,^ ^30^ ^,^ ^31^

Laser etching with the parameters that were used in this study significantly increased bracket shear bond strength using SBU. Laser etching increased surface roughness and created a surface with microretentive characteristic and microcracks that was favorable for resin bonding.^12^ ^,^ ^14^ ^,^ ^32^ This surface with microroughness was also evident in TXT with laser etching. However, laser etching did not increase shear bond strength for TXT. This result supported a previous report that the effect of laser etching depends on the type and compositional characteristics of adhesive resins.^9^ ^,^ ^12^ SBU, when compared with TXT, has lower viscosity due to its water and ethanol solvent content and the low viscosity monomer HEMA. The surface irregularities and roughness produced by laser etching may be wetted in a better way by SBU than TXT. Wetting the prepared enamel surface is considered a critical step in enamel bonding.^9^ The higher wetting ability of SBU relative to TXT on etched enamel could also explain the higher bracket-bonding of etch/SBU group, compared to that of etch/TXT group. These results revealing a significant difference between etching modes rejected the null hypothesis.

Some authors demonstrated that higher shear bond strength values are associated with high amounts of remnant adhesive on enamel surface.^1^ ^,^ ^6^ ^,^ ^13^ ^,^ ^24^ This was not necessarily observed in this study; no statistically significant difference was found when the ARI values were compared between the groups. Although no cohesive fracture in enamel was observed, there were enamel cracks in some groups. Enamel cracking is a serious problem during bracket debonding, compromising the intact surface of tooth. A slight relative relationship was found between high bracket bonding and enamel crack so that the laser/SBU group with the highest strength (15.38 MPa) and SBU/etch-15 with high strength (11.50 MPa) exhibited enamel cracks. However, crack formation was not observed for SBU with 5 and 10 s pre-etching with similar shear bond strength to that of 15 s etch group and for laser/TXT with low shear bond strength (7.14 MPa). In this regard, it has been suggested that bracket shear bond strengths over the fracture strength of enamel (approximately 14 MPa) are not desirable.^13^ ^,^ ^20^ ^,^ ^23^ Although bond strength was not high, enamel cracks were also seen in the etch/TXT group. The only possible explanation for this is that the longer etching time in this group created porous structure that might not be fully penetrated by resin. This may weaken the enamel surface and rendered it susceptible to crack formation. It can be concluded that the ARI score and occurrence of cracks seem to be dependent not only on the shear bond strength, but also on many factors such as adhesive composition, bracket base design and characteristics of the prepared enamel.^33^

Based on the results of the current study, the laser etch/SBU combination, etch/TXT and SBU/etch-15 should be approached with caution, as enamel cracks were observed. However, SBU in SE mode or with shortened pre-etching time (5 s) can be considered a safe treatment, with reduced chair-time and low risk of moisture contamination.

It is important to mention that a minimum bond strength of 6-8 MPa has been declared as adequate for most orthodontic needs during routine clinical use.^34^ Mean shear bond strength values of all groups in this study either fell in the mentioned range or were considerably higher. Having higher values however renders a safe margin that may protect against bracket debonding in the event of an unexpected trauma to the bracket-enamel interface. In this regards, SBU with 5 s etch (11.15 MPa) could provide this requirement with no enamel crack, while SBU in SE mode and laser/TXT had lower shear bond strength (7.53 and 7.14 MPa, respectively). 

In this study, 12 specimens were used in each group. A post power analysis using the mean and standard deviation values of the seven groups revealed a power value of 89% at α=0.05, which is deemed sufficient, as it is greater than 80%. As with other ex-vivo studies, it was necessary to rely on thermocycling to simulate the intra-oral conditions, which may differ to what may happen in a true clinical setting. This could be considered the main limitation of this study. The stress applied on the bonded bracket during debonding in clinic is a combination of shear, tensile and torsion forces. Also, enzymatic and pH challenge was not simulated in this in vitro condition. Therefore, randomized clinical studies are suggested to assess the performance of SBU in the clinical setting. Such studies will also give a better understanding of the feasibility of integrating such adhesives in routine orthodontic procedures. To evaluate only the effects of SBU and cancel possible confounding variables it was chosen to bond the brackets in all groups with a conventional orthodontic composite (Transbond XT). Following the results of this study, bonding orthodontic brackets with other types of available adhesives, especially those used in restorative dentistry, would be an interesting topic for future research, as it would be beneficial in reducing the number of items that are needed to be purchased in dental clinics.

## CONCLUSIONS

Based on the results of the present study, it can be concluded that:

1. Acid etching and conventional orthodontic adhesive can be replaced with SBU in SE mode without reducing bracket shear bond strength.
2. Adding an etching step before application of SBU improved the achieved shear bond strength. However, increasing the duration of etching from 5 to 15 seconds did not result in significantly different bracket shear bond strengths, while causing enamel damage in the form of cracks.
3. In contrast to the conventional orthodontic adhesive, laser etching had a significant impact on the shear bond strength achieved with SBU, although enamel damage was observed in a few of the specimens.
